# Entangled Assemblages: The Mutual Becoming of Food and More-than-Human Being

**DOI:** 10.1007/s10699-022-09858-w

**Published:** 2022-11-02

**Authors:** Wim Van Daele

**Affiliations:** Department of Nutrition and Public Health, University of Agder, Postbox 422, 4604 Kristiansand, Norway

**Keywords:** Food, Eating, Assemblages, Entanglement, Becoming

## Abstract

Food and life are intimately entangled. To grasp the underlying complexity of this seemingly simple statement, this article first introduces the approach to food/eating as an assemblage enacted by various heterogeneous components, and further develops it by engaging with actor network theory and material semiotics. Thereafter the focus turns to ‘entanglement’, as inspired by quantum physics, to elicit the basic dynamics of the entanglement of food and more-than-human beings, conceived of as involving mutual and differential becomings within and among assemblages. The article illustrates these entangled becomings by drawing upon examples from Sri Lanka, which in an intercultural philosophical fashion serve to establish an articulation (in the sense of a connection) between the proposed abstract approach to food and some basic premises of Buddhism and Ayurveda, a South Asian health system. Overall, the article crafts a conceptual toolbox and performs ontological groundwork wherein food and human beings as entangled assemblages provide a productive, refined, and sensitive research apparatus for the intimate study of more-than-human life and organization while also spurring novel theorizations through food/eating.

## Introduction

Food and human life are intimately entangled. This statement, which appears so simple, turns out on deeper reflection to be much more complicated than it seems. First, the ‘human’ is never exclusively human, for we are profoundly shaped by our external environments and internal microbial communities, thus troubling the neatly manufactured boundary between the human and non-human. Illuminating in this regard is the interplay between the gut microbiome and mental disorders via the gut-brain axis (Valles-Colomer et al., 2019). The notion of the more-than-human stems from the growing recognition that the human and the non-human are not neatly distinguishable, and foregrounds the co-constitutive entanglements of human life and sociality with other-than-human entities (Lien & Pálsson, 2021), which may include microbiomes, food, and spirits.

Second, I have elicited elsewhere (Van Daele, 2018a), food is not just food, but is rather an assemblage emerging as an ongoing arrangement of heterogeneous components that is continuously forming a new arrangement/assemblage with the eating human being. Other phenomena can also be regarded as assemblages as in the famous example of Hans riding a horse (Deleuze & Guattari, 2009), or the case of cities (e.g., Kamalipour & Peimani, 2015). Here we focus on food and human beings as entangled assemblages as productive for further research on more-than-human life as well as for novel theorization through food/eating and digesting (Mol, 2021; Paxson, forthcoming; Remotti, 1999; Van Daele, forthcoming). To realize this theoretical potential, however, we first need to craft a solid conceptual toolbox and perform the necessary ontological groundwork, so that we can elaborate a refined and sensitive food-based approach to the study of more-than-human life and organization. Food scholars have already demonstrated that food provides an apt *looking glass* or *window* into society, culture, the mind and so forth (e.g., Counihan, 1999; Counihan & Van Esterik, 2013); yet, while agreeing with the idea behind this statement, I find that such optic conceptualization renders food quite passive, neither affected by nor affecting its surroundings. Here, therefore, I wish to pay closer attention to the qualities of food and so propose a more agential, relational, intimate, and vibrant conceptualization of food/eating with which more-than-human beings are *entangled* as assemblages where both are implicated in mutual transformation and differential becoming (Barad, 2007). This groundwork requires conceptual-ontological crafting that foregrounds the multiple modalities of relations (Strathern, 2020), and renders cooking, food/eating, and digestion as productive assemblages to work with in philosophy (Mol, 2021), anthropology (Paxson forthcoming), and intercultural philosophical anthropology I pursue in this paper. Throughout this article I will refer to food and eating as food/eating, inspired by the Dutch and Flemish word *eten* which covers both food and eating simultaneously.

This paper begins by introducing key aspects of the suggested assemblage approach, which it then further refines through an exploration of cognate approaches: actor network theory and material semiotics (Beetz, 2016; Latour, 2005; Law, 1999, 2009; Mol, 2010). After showing how food/eating and digesting are enacted by a network or assemblage of heterogeneous components with which they are entangled, this article focuses on the nature of such entanglements from a quantum physics perspective. It illustrates these entangled enactments of cooking, food/eating, and digesting by drawing upon examples from Sri Lanka, based on a total of three years’ fieldwork. In addition, as part of our conceptual-ontological groundwork, these illustrations also serve to establish a cross-cultural articulation, in an intercultural philosophical vein, between the proposed conceptualization of food and some key ontological premises of Buddhism and Ayurveda, which is a South Asian health system prevalent in Sri Lanka. Indeed, our aim is to establish a dynamic and integrated (though not necessarily harmonious) conceptual-ontological framework that is open to ontological and cultural variation, so as to enable sensitive and situated research and further theoretical musings through/with cooking, food/eating, and digesting. The proposed framework does so by articulating (in the sense of connecting) food and its corollaries with the quantum-inspired notion of entanglement as well as assemblages and their cognates in the context of the study of more-than-human life and organization.

An important note is warranted here. Transporting, translating, and articulating quantum-inspired concepts into conceptual-ontological work where humanities are involved raises the question of metaphorical analogy versus material-ontological continuity. Our work here situates itself within the second category in line with Barad’s (2007:23) remark that issues of intentionality, meaning and matter “cannot be resolved by reasoning analogically; they require a different kind of analysis.” Here, she refers to a new science of mattering and agential realism that moves away from analogical representationalism undergirded by clear object-subject, matter-meaning dichotomies. Food/eating lends itself very well to exploring such fundamental onto-epistemological questions in novel ways, as it troubles the neat dichotomies undergirding much of our established conceptual apparatuses (Mol, 2021).

## Assemblages and Food/Eating

**Fig. 1:**
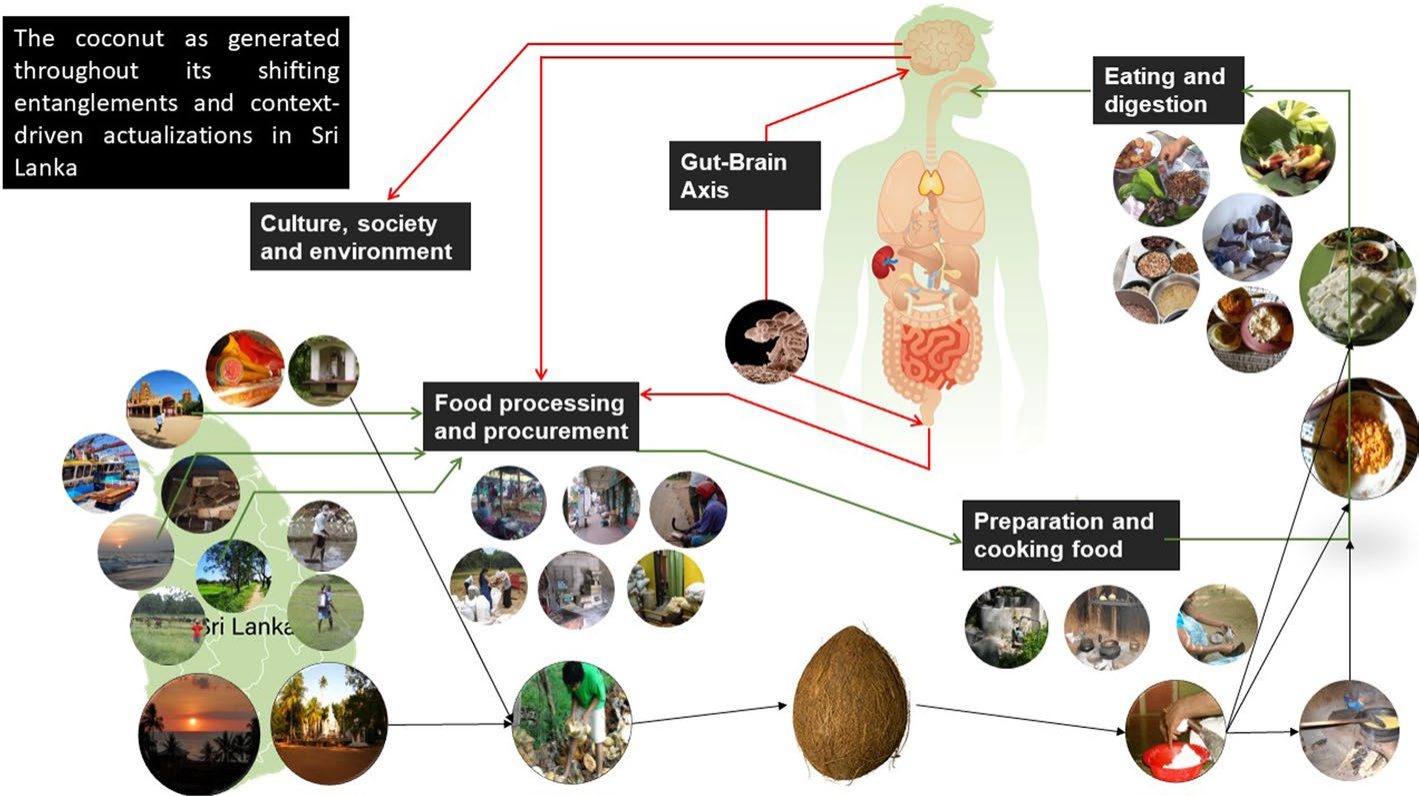
Visualization of the food and coconut assemblages throughout their entangled lifecourses in Sri Lanka. Pictures from author and drawing modified from Thomas Eikeland Fiskå

In line with DeLanda (2006, 2016) and Deleuze and Guattari (2009), and drawing on my observations of the diverse forms of food and the many roles it plays in various contexts, I hold food to be an assemblage that is an emergent, and not-necessarily harmonious whole of heterogeneous components that are themselves emerging or altering within this network of components. A particular food emerges in a particular form resulting from the conjunction of its component parts acting on each other in resonant or dissonant ways. Take the example of the coconut, which could be labeled as the second staple food after rice in Sri Lanka. In the suggested approach, the coconut is a resultant expression of the conjunction of sunshine, soil nutrients, water, human relationships, harvesting efforts, taste aspirations, market prices, deities, mythologies, ideas about health and so forth. Moreover, the emergent wholes are not reducible to the sum of their parts (as in a mixture), but the parts that act on each other are rather creating a new resulting expression (as a new entanglement), open to ongoing transformation (DeLanda, 2006:4–5, 2016: 19–21), as in the breaking and grating of the coconut so as to prepare coconut milk. The parts are not effaced into a seamless whole, but continue to exert their (partially altered) actions within the heterogeneous assemblage (Van Daele, 2018a). The agency *of* the assemblage is thus emergent from the parts while being different from any of the parts alone (Bennett, 2010:24). As such, the coconut (or any other food, for that matter) is “a continuously generated effect of the webs of relations” (Law, 2009:141) that alters throughout its lifecourse. In other words, the food gets enacted in the assemblage of components that co-constitute it, with eating as its teleological purpose (Fig. 1).

When component parts are detached from the whole and “plugged into a different assemblage” their interactions become different (DeLanda, 2006:10), creating a new expression of the affected assemblages. For instance, the interplay of heterogeneous forces and components form an evolving assemblage: the serrated iron resists the coconut flesh as it is moved back and forth by the hands, whereby the surface gets scratched and raw grated coconut emerges. The coconut turned into grated stuff is then mixed with water and squeezed, and thus further transforms into coconut-milk, which forms the basic ingredient in cooking numerous curries. Hence, the coconut in conjunction with added components acquires different expressions as it *becomes* different and thereby enacts different roles. The fact that these novel interactions change the actions of the components renders thus *both* the assemblage and its components emergent. Hence, there is nothing intrinsic to the assemblage that determines in a singular causal way the shape it will become (DeLanda, 2016:19–20). Rather, its ontological co-constitution emerges as a resultant from the ongoing multiple and complex interactions of the components within the assemblage. In other words, the assemblage is continuously being made, re-made, and sometimes unmade within the heterogeneous networks that enact it in different ways according to its various interactions and contexts. In this way, assemblage theory provides us with a non-essentialist, non-static, and non-linear approach to emergent entities (Van Daele, 2018a).

## Assemblage and Cognate Approaches

An assemblage approach is neither new nor unique in its sensitivity towards heterogeneity, multiplicity, and human/non-human entanglements. It resonates with Gottfried Wilhelm Leibniz’ *Monadology,* originally written in French in 1714 (1898, paragraph 6) where he contrasts the monad (in our words component), regarded as a simple substance, with the compound (in our words assemblage). More recently, the assemblage approach has found cognate ways of thinking in actor network theory and material semiotics, both of which focus on the emergence of entities resulting from heterogeneous relations, human and non-human, composing a network where agency is distributed (Latour, 2005; Law, 1999, 2009; Mol, 2010). In fact, Law (1999:4) remarks that “actor-network theory may be understood as a *semiotics of materiality*. It takes the semiotic insight, that of the relationality of entities, the notion that they are produced in relations, and applies this ruthlessly to all materials—and not simply to those that are linguistic.” Moreover, the entities *emerge* from the relations in which they are situated and thus are “*performed* in, by, and through those relations” (Law, 1999:4). So, although Law (2009:142; 1999:10) advocates speaking about material semiotics rather than actor network theory^1^ (sometimes represented by the acronym ANT), he also stresses that there is not *one* actor network theory since it is a diaspora drawing upon other intellectual traditions and proliferating in many directions, which goes for assemblage theory as well. The actor network theory has multiple roots from which it has diverged and proliferated. Most notably, it draws upon Michel Callon’s materially heterogeneous systems, borrows from Thomas Kuhn and the sociologists of scientific knowledge, is inspired by the notion of translation that is both about equivalences and betrayal or shifts, and is grounded in poststructuralist thinking of relational effects and Deleuze’s philosophy (Law, 2009: 142–146), the latter source being all-too-often left untreated in present-day theories of assemblages and their cognates (Buchanan, 2021). Actor network theory is continuously moving into different terrains, such as Cultural Studies and Science and Technology Studies (STS), and “as it has spread it has translated itself into something new, indeed into many things that are new and different from one another. It has converted itself into a range of practices…Its parts are different from one another. But they are also (here is the point) partially connected” (Law, 1999:10). This means that it is very difficult to fix it in a single definition; rather, it is a singular plural (Nancy, 2000) comprising some ingredients it shares with the cognate assemblage approach proposed here, including the sensitivity towards relationality, heterogeneity, materiality, process, non-linearity, and emergence. Given this multiplicity, Mol (2010:261–262) therefore suggests approaching the ‘theory’ of ANT^2^ rather as a repertoire “of terms and modes of engaging with the world, a set of contrary methodological reflexes.” I surmise that this repertoire and the process of food/eating can speak to and mutually enrich each other because of their shared heterogeneity and transience. Their trans-action will aid in crafting a new conceptual-ontological engagement with the world, allowing us to study it in more intimate and visceral ways, and to shift our take on the studied realties and so enhance our intimate attentiveness to the fragility of existence, the phenomena of the world being so prone to destabilization and transformation. Moreover, this mutual enrichment of food/eating and assemblages enables us to rework our engagement with the world as Mol (2021) demonstrates eloquently when deploying eating to craft a novel philosophical approach to the established concepts of Being, Knowing, Doing and Relating.

To return to our examination of the network of inspirational sources and actor network theory, Latour’s (1993) account of the scientist Louis Pasteur is an illuminating example, showing that, while the dominant narrative singled him out as the discoverer of anthrax, it was rather the association of heterogeneous elements that co-produced the discovery. Bruno Latour places the microbe, laboratory instruments, hygiene concerns, and researchers on a single plane as they compose a network of actants where all actions become relational effects (Law, 2009:145) because the discovery is not reducible to any one of them. Rather, the agency in the verb *dis-covering* is distributed within the network of actors that only attain their capacity to act, affect, and be affected *within* that network of relations. Hence the ability or capacity of Louis Pasteur to act and discover only emerged within these very relationships. In ANT, agency is thus decoupled from human intentionality and gets distributed among human and non-human actants or actors, and as such the human subject becomes decentered (Latour, 2005:71). The focus in ANT on the relational emergence of a state of affairs in complex heterogeneous relationships between human and non-human entities makes it resonate closely with the assemblage approach. Law (2009:146) has argued that there is actually little difference between the cognate terms ‘actor-network’ and ‘assemblage’, the latter translated from the French ‘*agencement*’ which refers to ongoing arrangement and re-arrangement. Actually, the notion of *agencement* seems to be better suited to evoke the relational *agency* that is ongoing within the ever-emerging and ever-becoming assemblage and its parts through ongoing arrangement (Ingold, 2020:22). An important difference between ANT and assemblage theory, however, is that the former flattens the topology into the form of the network (Law, 1999:6) whereas in the approach suggested here, agency maybe distributed unevenly according to the networks and contexts, whereby relational capacity can vary in terms of the power to influence or affect, according to circumstances or contexts of the conjunction of entities, and hierarchies may emerge among the parts of the assemblage and between assemblages themselves. For instance, a microbe may wreak havoc in an already weakened human body, but at other times could be destroyed by the healthy body’s defense system. This context-driven dynamic of the power to affect, resonating with Foucaultian power (Prado, 2018), leads to a more fluctuating or ‘bubbling’ topology of empowered parts and assemblages, which constitutes an important reason for turning to assemblages while remaining resonant with ANT with respect to its repertoire of relational agency, materiality, and enactment. This heightens our attentiveness to the need to carefully engage with the vibrant components, both in their relation to each other and in relation to the whole of which they are parts-in-the-making, as in the case of a symphony (Bohm, 1980:52) or rather polyphony (Van Daele, 2018a). And, wedding this repertoire with cooking, food/eating and digesting offers us a more fleshy, visceral and sensorial conceptual-ontological engagement with the world, and as such enhances our attentiveness to life as entangled and transforming.

## Relations of Mutual and Differential Becoming

Assemblages can exchange parts whereby both the assemblages and their parts alter. While exchanging components, the assemblages form an *interassemblage* until the cut is enacted, through which they *emerge* as differentiated and transformed assemblages that have until then been engaged in mutual transformation and *mutual becoming* (Deleuze & Guattari, 2009:242–243, 306, 337). Thereafter, they continue to perform mutual becomings that are enacted in other relations. For instance, both food and eater mutually transform when both constitute an interassemblage during digestion, where components are interchanged until the anus enacts the cut with food-turned-into-stool (we return to this later). This example illustrates, moreover, that component-parts can be exchanged between assemblages of varying scales (DeLanda, 2006:33–45), and thus assemblages also affect and are affected by their respective contexts (food in human body). In discussing these part-to-assemblage or micro–macro relations, DeLanda (2006) regards human bodies as component-assemblages of larger organization-assemblages that include wires, computers, ideas, the ethos, and so forth. In their turn, organizations are components of national or global assemblages (see also Ong & Collier, 2005). Yet, contrary to DeLanda, who apparently regards the nature of these scales as pre-given, I do not regard them as quite determined, since in this exchange of components across scales, all the parts, assemblages, *and scales themselves* transform in this process of mutual or relational becoming. The scale of a Corona virus at a market in Wuhan is very different from the scale a Corona virus acquired when it was aided by airplanes in its globalizing reproduction, creating worldwide disruption. Hence, scale itself shifts since the size, magnitude, and scope of agency in an emergent state of affairs are all parts of its becoming. We thus have a bubbling topology of entities engaged in the ‘cooking’ of life (Van Daele, 2013), unlike the flat topology of ANT.

As already hinted at, the process of mutual becoming can be clarified by an illustration that considers the digestive process in terms of assemblages. When I receive rice on my plate, I have an assemblage, which is a resulting and ongoing expression of the components and forces of eight amino-acids, complex carbohydrates, oil, bran, fire-heat, cooking effort, smell, market dispositions, consumer tastes, deity protection, gendered divisions of labor, etc. On the plate, this complex assemblage forms an interassemblage with the adjoining curries made on the basis of the coconut-milk prepared earlier. This interassemblage—which is thus an assemblage itself—constitutes the meal, and during the act of eating includes additional behavioral and moral codes. Moreover, the fingers of my right hand become part of the assemblage of rice and curry and interact with the other components to turn it into a small ball to bring to my mouth. By then the finger actions have entered the food assemblage and disrupted it to such an extent that it already looks as if it is partly digested even before entering my mouth. The conjoined forces of the actions of my fingers acquire more of the distributed agency enabled within this meal-related network of relations and form an *operator* or *assemblage converter* (Bennett, 2010:42) causing a drastic alteration in the food, catalyzing further transformation. Upon entering the mouth, the food ball enters a moment of intensive exchange with the mouth machine. The mouth microbiota aid in preparing the food as it becomes moistened by saliva. The forces of the teeth and the tongue turn it around, transform it further, and bring it to the edge of the digestive tract. In exchange, it releases heat, taste, and the love or dedication of the cook,^3^ all of which affect my mood, experience, and memories.^4^ As it goes down the tract, I get more enmeshed with the food. My gastric juices work together with the gut microbiota to make the messy substance release its nutritional components and absorb the micro- and macronutrients and energy. While this process takes place, my body gets fed, and my memory flies off to previous days. After my ‘digestive nap’, the food has in a fascinating alchemical way also transformed into ideas, some more silly than others, as well into energy to help me get on with the day (Châtelet, 1998:39). Later, I must release myself from the food I ate. The anus machine enacts the cut between myself and the by then abject food-turned-into-excrement. Noëlle Châtelet (1998:40) puts it succinctly: “manger, c’est se connecter, au delà de connections intermédiare, la bouche et l’anus” [to eat is to connect, apart from intermediate connections, the mouth and the anus]. Thus, throughout the interaction of the food ball and myself, we both pass through several states of transformation, or rather we both transform continuously in fascinating ways. We are implicated in a mutual becoming: I turn into an energized and ‘inspirited’ person, a human becoming (Ingold, 2013) ready to create, write, and become entangled with other materials and work; whereas the food-turned-into-excrement, cut away from our entanglement by the anus, continues to interact with other bacteria and goes on performing new becomings. Indeed, emergence, transformation, and becoming are part of the repertoire of our assemblage approach.

Hence, the process of digesting food is full of mutual or differential becomings and it is this that spurs our experimentation with deploying food/eating as an onto-epistemological approach to the study of more-than-human life and organization. What different work would the ‘eating’ and ‘digesting’ of our subject of research do, in place of ‘viewing’ and ‘clarifying’ it? We would at least have a more intimate rendering of our research subject and our manner of relating to it, where the researcher is both affected and affecting as well. Hence, this briefly illustrates how food/eating and its corollaries are good to work with in crafting novel onto-epistemological approaches (Mol, 2008, 2021; Remotti, 1999; Strathern, 2012) and why this groundwork is necessary to be able to perform further such work.

## A Cross-Cultural Excursion: Engaging Assemblages with Ayurveda and Buddhism

Another reason for deploying assemblage theory in our conceptualization of food/eating lies in the comparative and cross-cultural potential opened up by combination of the radical abstraction of the former with the visceral implications of the latter, a fertile conjunction in terms of generality and specificity. The theoretical frame must be kept sufficiently abstract to enable its circulation across different contexts and allow it to be modified according to the research area with which it is to be articulated (connected) and enfleshed in conjunction with food/eating. To illustrate, let me explore how the abstract frame can be modified to resonate and articulate with Buddhism and Ayurveda, a South Asian system of health practice,^5^ and thereby facilitates cross-cultural comparison.

Ayurveda draws upon Indian Samkya philosophy, in which primordial matter (*prakrti*) has evolved into the five proto-elements or *pancabhūtas* (water, earth, fire, wind, and ether) that permeate the entities populating the world (Obeyesekere, 1977:155–156). In Ayurveda all entities are made up of these proto-elements as well as the three humors (wind, bile and phlegm), but in different combinations accounting for the differentiation between entities. In the human person, these basic elements and forces are continuously shaping the seven body elements or *dhātus*—food juice or nutrient fluid, blood, flesh, fat, bone, marrow, and semen (Obeyesekere, 1998:201) whereby the body is in an ongoing state of flux. Additionally, all entities contain the qualities of *rasa* (flavour), *guna* (beneficial quality and healthy), and *dōsa* (absence of beneficial quality and state of ill-health) (Seneviratne, 1992:179–184). By contrast, in the Buddhist view the human person is composed of a particular combination of the five aggregates: those of materiality, feeling, perception, formations, and consciousness (Buddhaghosa, 1976:489). The materiality aggregate includes four instead of five proto-elements: earth, water, fire, and air. In both Ayurveda and Buddhism all entities are thus composed of identical elements and aggregates distributed in specifically individuated ways. When assemblage theory is articulated with both of these South Asian systems, they come to resonate in that entities are emergent entanglements and differentiations of such heterogeneous elements. We then also see a clear resonance with the ever-emergent, dynamic, co-nascent, and transformative nature of assemblages in Ayurveda and the Buddhist notion of the impermanence of matter and life.^6^ Such an articulation creates novel openings for the study of more-than-human life and organization, as well as their evolution in the short or long-run. For instance, it would be fascinating to study how these Asian systems of thought and practice are written into the situated (micro)biology of Sri Lankan eating bodies and their guts via their food rules that are informed by elemental and humoral theories. Such a study would fit well within the agenda of decentering the universalized biology of the human body and highlighting the decentered bodies’ situatedness as entangled with their microbiosocial environments as a clear instantiation of the entanglement of food and more-than human becoming (Niewöhner & Lock, 2018).

To return to the example of eating/digesting, albeit here rendered in Ayurvedic terms, the fire element plays a central role in digestion and hints at how the assemblage theory and its cognates could be articulated more closely with this Asian health system in an intercultural philosophical fashion.^7^ I eat the rice with the curries which are transformed by the digestive fire into the seven *dhātus* of the human body (Obeyesekere, 1998:201), which the five basic elements continue to permeate. The seven bodily *dhātus* re-combine into the three bodily humors that in Sinhalese are termed *va* (air), *pit* (bile), and *sem* (phlegm) (Seneviratne, 1992:180), and which need to be balanced to maintain both good physical and mental health. Like the five elements and the three humors, the *dhātus* remain in a constant flux according to the heaviness, hotness, *rasa*, and *guna* of the food swallowed (Seneviratne, 1992:180–181). The balances and components composing the human assemblage thus alter conjointly with the food being cooked and transformed by the gastric fire (resonant with the biomedical notion of metabolism), further turning food into excrement and finer waste (e.g., hair, nails). Enfleshing a mutual becoming, the person gains strength and (mental) health, whereas food is turned into waste. Additionally, food feeds the gastric fire and thus upholds the good health and life of the devouring person. Hence, in the digestive inter-action between the assemblages of food and human being, or intra-action within the eating/digesting human being, the gastric fire cooks this process of mutual transformation while nourishing itself.

In Sinhalese, the terms for kitchen (*kusiye*) and stomach (*kuse*) are closely related, linking the hearth-fire in the cooking of food with the gastric fire of digestion, extended into the overall ‘cooking’ of life that includes other processes of transformation and mutual becomings across the various realms of life. Indeed, ‘cooking’ among Hindus and Sinhalese encompasses “all those conditions and stages that help raise crops in the field, including ‘ripening’, and those which help its actual procurement and handling up to the actual ingestion” (Khare, 1976:3). In a similar vein Seneviratne (1992: 190) argues that for Sinhalese people “there is no distinction between human and natural causation in the process of food transformation from raw to cooked, since both are said to involve ‘ripening’,”^8^ while Malamoud (1996:48) has found that according to Vedic texts, the sun ‘cooks’ everything in the world.

In sum, the transformative and life-sustaining role of the gastric fire resonates with the role of the log-fire employed in the actual cooking of the food and the sun-fire required in the growing, ripening, and ‘cooking’ of food-bearing plants. Sun-fire, hearth-fire, and gastric fire become assemblage converters that transform the assemblage of food throughout its entire life cycle—cultivation, distribution, preparation, and consumption—where it exchanges components with the human assemblage, and where both are implicated in an ongoing mutual becoming in the process of ‘cooking’ by various fires. However, the transformative and regenerative fire is ambiguous and must be carefully managed throughout the various cycles of cooking food and ‘cooking’ life. For instance, ritual heat must be contained by sprinkling water on the spirit priest, so as to cool him down and so avoid him becoming devoured and destroyed by it (Tanaka, 1997:129–150). The alternation of these ambiguous processes of heated transformation, on the one hand, and the (likewise ambivalent) cooling stabilization, on the other, both embodied in the allegorical ‘cooking’ and actual cooking, infuses assemblages with a more dynamic distribution of agency involving both accelerated (heating) transformations and decelerated (cooling) stabilizations which account for more enduring phenomena. This alchemizes assemblages by foregrounding the bubbling pulsations of life in its ‘cooking’ as entangled with the ongoing regeneration of the world.^9^

## Entanglements

Returning to the conceptual-ontological frame, it is still necessary to further clarify the nature of the relations and boundaries of assemblages and their components, as well as to engage with questions regarding emergence, transformations, and causality. To recapitulate, an assemblage emerges from components acting on and transforming each other. When assemblages interact components get exchanged, and both components and assemblages are implicated in a process of mutual becoming. Thus, entities are continuously generated and enacted in a network of inter-acting and trans-acting elements, whereby entities lack an independent, self-contained existence. Rather they emerge as relational effects in what we call ‘entanglements’ in line with quantum physics (Barad, 2007; Steinmo, 2017).^10^

Involving quantum physics into our frame entails that we widen our focus from considering entities as distinct objects, localizable at a single point in space and time, as classical Newtonian (social) science would have it, so as to include the relational processes of becoming through which entities as effects emerge and individuate (Barad, 2007: 97–107, 137–138, 250–252; Trnka & Lorencová, 2016). Quantum physics indeed teaches us that entities (re)emerge from an undifferentiated whole in a process that Barad calls (2007:332–333) ‘differential becoming’. In short, entities differentiate and emerge *through and from* a fundamental relationality where nothing is distinguishable and that is thus ontologically prior to discernible entities and phenomena. Bohm (1980:188) describes this undivided reality or whole as the *implicate order*—derived from the notion *implicit* where something is not clear because it is enfolded, which is designated by the verb ‘to implicate’. This realm is the vortex of becoming from where new wholes and entities continuously unfold, emerge, and actualize their potential (Rosado, 2008:2080–2081; Gabora & Aerts, 2005). In contrast, Newtonian physics studies the observable effects of such emergence; the apparently discrete entities that dwell in the *explicate order*—where something is made *explicit* and folded outward, acquiring clear discernible boundaries (Bohm, 1980). In other words, there are two sides to reality: one is the realm of sheer potentiality where all is enfolded and nothing has yet been differentiated or unfolded or actualized; and the other realm of actualized and observable entities—with both domains being enmeshed as a whole (Trnka & Lorencová, 2016:30).

What is more, entities remain related after their actualization and apparent separation, since they never become entirely self-contained and independent: this is the state of entanglement that quantum physics refers to and from which we draw here. The phenomenon of entanglement explains how particles operate as a single entity or whole, even after their separation and at a large distance, something that Albert Einstein branded as “spooky action at a distance” (in Steinmo, 2017:196; Vedral, 2011:41). Hence, the focus is less on entities per se, but rather on the entangled dynamics of the agencies that shape them (Barad, 2007:23). The notion of entanglement can be further clarified, by way of example, through discussing what in quantum physics is called the observer effect (Trnka & Lorencová, 2016: 16, 19). A particular measuring apparatus does not merely measure ‘a reality out there’, but also contributes to shaping that reality. The entanglement in this observing event or phenomenon consists of the fact that the object (e.g., a specific wave behavior) only comes into existence *through* interaction with the measuring apparatus (in this case, a particular set-up of the two-slit experiment) made to observe it. Thus, the observer effect entails the one-ness of observer and the observed, both remaining entangled. An example of the observer effect in human sciences is the case where interviews shape the answers of the respondents, or where anthropologists incite certain expectations among their hosts. As such, they affect the whole of which they are part and which they seek to study. Hence, ‘entanglement’ as here conceived does not refer to a relation between discrete and distinct entities—the observer, the observed and the measuring instrument—that remain largely unaffected by that relationship, but it rather emphasizes how these enmesh, emerge, and differentiate out from an undivided whole (e.g., the interview event) (Barad, 2007: 308–340; Bohm, 1980: 163–169). In other words, an entanglement is different from a relation established a posteriori between individual relata. It is instead a differential becoming, prior to these individuations with which (social) researchers engage, that takes place between trans-acting assemblages and components (e.g., interviewer, interviewee, words, ideas) within whole phenomena (the interview setting). The differential becoming entails thus a joint unfolding and actualization from the implicate order of the whole phenomenon from which the renewed entities emerge (interviewer with new information, respondent thinking differently because of the unexpected questions).

As already pointed out, even after entities have differentiated, they remain entangled—in the sense that they continue to be affected by the intense exchange of components that occurred during the time they were part of the whole interassemblage (recall Einstein’s “spooky action at a distance”). Hence, they never become completely separated and exterior to each other, and as such a mutual becoming is always taking place and never-ending, even though this may involve both accelerated transformation and stabilization. For instance, the strange questions of the interviewer may continue to stir further musings among respondents long after the interview event. Or, to return to food, when ingested, it co-constitutes an interassemblage with the human body during which an intense process of exchange and mutual becoming takes place (Karen Barad calls this the ‘intra-action’ within the whole phenomenon); but the process of mutual becoming does not end even when both have differentiated and separated, as the digested food continues to affect the person and their microbiota, in turn affecting the digestion of the next food as well as the stool’s ongoing engagements elsewhere. Hence, even though they appear to have separated, they remain entangled since the effects of their entanglement persist after differentiation. In sum, during the entanglement of assemblages and components, these elements form an undivided whole wherein entities have no boundaries. The entities, and their boundaries, only emerge after the differentiation, individuation, and particular actualization of potential becomings, but still remain permeated by that very relationality that continues to shape them, and thus they remain entangled and continue to be enacted by the shifting networks in which they may find themselves after the enacted cuts.

Since entanglement entails a mutual becoming and differentiation that takes place within and between assemblages, it is not a relation between self-contained and discrete entities, and this has a bearing on the nature of causality. In classical physics the cause becomes separable from the effect in space and time within a mechanistic scheme of singular determinist relationships between individual components (Barad, 2007:66; Mol, 2010:261). Causality in the proposed assemblage approach is very different, since it is *itself* implied in the process of differential becoming where causality becomes and remains an entangled affair (Barad, 2007:174–179). Hence causalities can only be traced afterwards: as effects, as they emerge and actualize in a complex network of distributed agency (likewise in actor network theory and material semiotics). Causality in the implicate order is still full of endless possibilities until it unfolds and actualizes in the process of differential becoming, where specific causalities can be traced a posteriori. This multiplex and undetermined causality stands in stark contrast with the singular determinist relationships that are prevalent in Newtonian (social) sciences.

## Qualifying ‘Emergence’

The conceptual repertoire of ANT and assemblage theory cultivates a sensitivity toward multiplicity, heterogeneity, non-linearity, relationality, materiality, process, and emergence. In focusing predominantly on undivided phenomena, as the primary ontological unit from which entities differentiate, Barad (2007) heavily emphasizes the process of emergence and differential becoming, something shared by ANT, assemblage theory and cognate approaches, but which seems to acquire an even more radical tinge in reference to ‘entanglements’. If we understand her correctly, it is impossible to speak or write about something until after it has differentiated itself from an undivided whole; that is until after the moment of relational *origin*-al becoming whereby it becomes part of the explicate order. Bohm (1980) acknowledges that it is indeed impossible to write about the implicate order—the vortex of becoming—and thus research about more-than-human life seeks to describe the unfolded order as well as the a posteriori tracing of the unfolding and actualization of events, drawing attention to the processes of their becoming. The realm of the implicate order is also impossible to write about, as by writing about it we have then already contributed to explicating the example into the explicate order. Hence, all examples given here necessarily dwell in the latter order, but remain enmeshed with the former.

An important note of caution: if we uncritically deploy ‘entanglement’ and ‘differential becoming’ in accounting for all phenomena, we would be in danger of placing an excessive focus on their ongoing generativity and leaving insufficient space to account for enduring entities and their historicity either before or after their ‘implication’ in the vortex of becoming. DeLanda (2006:38) succinctly re-phrases this concern of a misplaced emphasis on relentless becoming and emergence: “It runs the risk of placing too much emphasis on the historical birth of a particular assemblage, that is, on the processes behind the original emergence of its identity, at the expense of those processes which must maintain its identity between its birth and its death.” Such an undue focus on emergence moreover lead us to adopt a one-sided approach to process, where it would be always transformative and creative, whereas process and becoming are equally involved in the maintenance of stability. For instance, the flame of a candle may seem static when there is no wind, but nevertheless remains an ongoing process until extinguished. Likewise, continuous stirring may be necessary to hold the separate ingredients together in a sauce so as to avoid separation. Both the emergent and the stabilizing processes conjoin in the ongoing becomings and enactments within networks or assemblages.

Deleuze and Guattari (2009, 2011), who are foundational to the repertoire of concepts under the heading of assemblage theory even though they remain all too often unacknowledged (Buchanan, 2021), also stress *both* these emergent and stabilized aspects in their approach via the respective notions of *deterritorialization* and *territorialization*. When assemblages get entangled into an interassemblage, they embroil with each other and exchange components while they are trans-forming and trans-acting as part of the vortex of becoming. The assemblages then move, transform, and destabilize in their entanglement, and this process is referred to as deterritorialization. Thus, to continue with the eating example, when my hand moves to bring the food to my mouth, there is a double spatial movement or deterritorialization. The displacement of the hand in space and time effects another deterritorialization of the foodstuff. Thereafter, when the teeth and tongue move the food ball round and round, all deterritorialize while mutually transforming and destabilizing; a process continued during digestion. Transformation and deterritorialization are thus bound up with each other and extend the initially geo-spatial connotation into a broader notion of destabilization (DeLanda, 2006:12). The fact that some entities can move, transform, and destabilize (all pertaining to Deleuzian deterritorialization) includes the possibility of its opposite as well: *territorialization* as the stabilization into enduring phenomena, which can become destabilized again later in a new entanglement. For Deleuze and Guattari (2009:291–294), the political project against total singularity and for multiplicity precisely seeks to destabilize and deterritorialize powerful territorialized and crystallized structures—which explains their emphasis on deterritorialization—but this also implies that they do explicitly acknowledge the existence of enduring and powerful processes, something which has not received sufficient attention since their political project of deterritorialization has often been confounded with their descriptions of both territorialization and deterritorialization (Patton, 2000:42–48). Thus, duration needs to be (re)included in assemblage theory to account for the oscillation between stabilizations and destabilizations in the ongoing processes of entanglement that crisscross the implicate and explicate realities of the world.

## Foregrounding the Generative and Creative Dynamics of Food/Eating

In *Anti-Oedipus* (2011), the first book of Gilles Deleuze and Félix Guattari’s two-volume *Capitalism and Schizophrenia*, assemblages are held to be desiring machines where desire is immanent (see also Buchanan, 2021). Phillip Goodchild (1996) explains that in *Anti-Oedipus* they give prominence to desire as an immanent creative force in the notion of “machine” to stress its machinic—generative and creative—quality but that they render desire less prominent in the second book *A Thousand Plateaus* (2009). In *Anti-Oedipus* (2011), desire is immanent to the functioning of machines or assemblages, and this again resonates clearly with ancient Vedic and Buddhist thinking where desire is a creative force that permeates the universe and its entities, and is thus inherently part of the interactions it creates and from which it arises (Webster, 2005:53, 137). Hence, in an intercultural philosophical vein and as part of our conceptual-ontological work, desire acquires a nearly ontological force as part of the vortex of becoming and thereby immanent in our assemblages. Desire, which has numerous forms as evidence in the many words for it (Webster, 2005), emerges from the sparks generated by inter-acting assemblages or components to which desire remains immanent and which it energizes, while enhancing further mutual becomings. As in the conjunction of food and human beings, desire emerges as both mutually desire and deterriorialize each other (Van Daele, 2016). We may indeed ask, if someone desires foods, who does the desiring and which form(s) does it take—lust, hunger, seduction, etc.? How do/does desiring foods move people? Cinnamon has clearly moved people across the world in their desire for spices, but where is that desire located? Does the food seduce the person to select it, or does the person solely desire that food? Shop keepers play with this ambiguity every day by showing the best sides of their fruits to seduce passers-by to choose food from their stalls or shop. The answer to where desire is situated is that it is in our frame being distributed and immanent in the network-assemblage of food as it entangles with the more-than-human becoming and expresses itself in numerous forms within multiple becomings.

Having thus re-incorporated desire from *Anti-Oedipus* as a creative force and stressed foods machinic—generative and creative—quality that is enabled by its entangled states, we could go further and conceptualize food, along with Deleuze and Guattari (2009:71, 88–90, 145, 333), as a *machinic assemblage* that produces a variety of other heterogeneous confederations and aggregations in life by passing through different assemblages and milieus, forming interassemblages, causing intermingling, and producing multiple becomings. In its life from seed to excrement and in its intricate embroilment with (human) life, food/eating is productive of relations of intimate sharing (taking over the role of the umbilical cord linking fetus and mother), microbial communities within, labor exchange, harvest rituals, markets, policies, relations with invisible phenomena, international trade agreements, and speculative financial arrangements. However, given that ‘machinic assemblage’ is a pleonasm—the two terms being similar—it remains sufficient to conceptualize food as an assemblage, while pointing out that it is a particularly powerful one. Food’s machinic capacity emerges relationally, and it is precisely because food is enacted by an exceptionally wide range of entanglements that it becomes such an intense and condense agent in shaping human and more-than-human life and organization (Van Daele, 2018b). This assemblage is continually in flux throughout its shifting entanglements and becomings as permeated by the creative forces of immanent desire, yet it also entails stabilizations. Food, like anything else, is an assemblage, but it acquires an additional intensity in conjunction with the eating human being as it passes through the gastro-intestinal tract and spurs intense mutual becomings, aided by the billions of desiring microbiota.

It becomes easy to understand how food becomes machinic or productive by returning to the lifeline of food from seed to excrement, in the process of which it enters into multiple relations involving the mutual exchange of components with other entities while performing differential becomings throughout its own life-course (which also blurs the boundaries of ‘food’, as its former becomings as plant or animal and other processes of transformation are all included in our conceptualization of food/eating), constituting a dynamic and complex food system.^11^ When the farmer takes out paddy seeds from the bag, the collection of seeds has already been engaged in a process of becoming through the components it has been collecting and shaping, including aspects of technological innovations (green revolution breeding techniques in specialized farms), intellectual property rights, packaging and storage techniques (without which the seeds may look and act differently), and the scientific and political aspirations—in this case—making postcolonial Sri Lanka self-sufficient in food. The farmer becomes part of a complex entanglement involving economic markets and agricultural extension officers who promote green revolution seeds, and thus becomes an added component in the maintenance of ideas and institutions that emerge around paddy seeds. The seeds, moreover, enact additional relations between seed-exchanging and labor-sharing farmers (bringing people together in closer friendships or at times causing friction) and their families. When the seeds are sown and nestling in the mud, they *adsorb*—add while preserving some aspects of their agential qualities that remain from former entanglements (Bennett, 2010:35)—elements from its new milieu: nutrients from the earth, water, sunshine, care, human effort, divine and chemical protection against pests, and so forth. While adsorbing, the food assemblage alters into a plant, which releases sweet scents when its pods fill with a milky substance that gradually hardens into paddy. Then the sweet smell of the paddy incites competition between elephants and human beings over who will consume it, spurring the latter to make various arrangements for protection (organizing watch-keeping) against the voracious appetite of elephants, and for the exchange of labor during the harvest period. Hence, food enters a milieu, adsorbs components, while releasing its own, and effects mutual transformations by engendering various arrangements in the milieu (relations of keeping watch) that condition food’s own resultant expression (as either elephant or human excrement) (Deleuze & Guattari, 2009:312–318). Food/eating is thus a machinic assemblage that in its entanglement with various other assemblages and milieus is *net-working* and effectuating mutual and multiple becomings that entail context-driven actualizations (Gabora & Aerts, 2005) and which becomes co-productive of various arrangements, aggregations, and assemblages that populate the world and more-than-human life.

## Conclusion

This article has thus itself been implicated in the entanglement of food/eating and more-than-human becomings with assemblages, Ayurvedic and Buddhist philosophy, Sri Lankan ethnographic engagements, and the quantum-inspired notion of entanglement so as to craft this particular ontological-conceptual arrangement. My aim in doing so has been to establish a solid foundation for future research work through/with food/eating in a way that will offer an alternative to dominant language-based models that are grounded in a human-centered ontology. The reason for deploying food/eating (and its corollaries of cooking and digesting) as an agential research device in the study of more-than-human life and organization is to increase our sensitivity towards the fleshy and entangled existing of more-than-human becoming, and thereby enable new modes of research that gets implicated in the ontological constitution performed by this research work. Yet, to enable this agential role, it has been necessary to first engage food/eating in a mutually reinforcing relation with the particular elaboration of the assemblage repertoire and entanglement. To stress the co-creative capacity of food/eating in shaping more-than-human life, and establish its conceptual-ontological potential as part of an agential research approach, I have specified that it is a *machinic* assemblage that is enacted through its entanglements with heterogeneous components, assemblages, and contexts in relations of mutual and differential becomings that involve processes of context-driven actualizations in the overall regeneration of the world and life in which our research partakes. Modified along these lines, food/eating offers us a refined and sensitive conceptual-ontological apparatus which will allow us to modify our (research) relationship with the world, both ontologically and epistemologically, as Mol (2021) has already suggested when examining the four key philosophical concepts of Knowing, Being, Doing and Relating, through food/eating. Additionally, our assemblage-inspired food/eating approach from the start in-corpo-rates the careful consideration of intercultural or inter-onto-epistemological sensitivity into its arrangement, by drawing upon Ayurveda and Buddhism; yet such pluralization requires further work through ongoing enrichments and entanglements across different contexts. However, the hope is that the conceptual groundwork crafted here can spur or contribute to further such entanglements and ontological co-constitution in future research.

## Footnotes

1 Different authors write “actor network theory” differently and I adopt the way of writing as associated with the referenced authors.

2 Actually, it was Michel Callon who, in the early 1980s, was the first to speak of *acteur-reseau,* and this term was later translated into ‘actor-network’ in English, turning into the famous acronym of ANT in the early 1990s (Mol 2010:254). Bruno Latour himself did not use ‘actor network’ until the end of the 1990s and it is therefore striking that he has been identified with this term, an identification he nevertheless subscribed to in his *Reassembling the Social* (2005).

3 Such as the spirit of the giver or *hau* of the food gift (Mauss, 1990:11).

4 Think of the famous madeleine de Proust where the sponge cake eaten by Marcel Proust brings him back to childhood memories.

5 Food plays a central role in Ayurveda, which thoroughly influences Sri Lankan food habits to date.

6 Which seems to contradict the apparently indivisible proto-elements as the most basic building blocks of the world in Ayurveda and Buddhism. Perhaps the nearly universal desire to identify the basic constitution or origin of the world in gods or particles, or quarks, may set the ontological constitution of proto-elements in tension with the law of impermanence in the Buddhist context.

7 The example opens up a more alchemical rendering of assemblage theory that I have introduced and elaborated elsewhere (Van Daele, 2013).

8 Anand Pandian (2011:159) moreover shows beautifully how the ripening of crops conjoins with the maturation of people in postcolonial India, stating “‘Ripening’ brings the development both of subject and of nature—or, both of self and landscape—into a common frame”.

9 For a more extensive discussion see Van Daele (2013, 2018b).

10 I am thus drawing upon insights from quantum physics to elicit dynamics of emergence and becoming (of both matter and meaning) that permeate phenomena across scales and, following Barad (2007:3–25), I do not use these insights in a merely metaphoric sense.

11 As such, our approach offers a refinement to current definitions and the hype around food systems, which tend to focus on the (human-centered) production, processing, distribution, and consumption of food and mostly its environmental, economic, and political contexts in which these activities take place. Our approach contributes to the understanding of food systems by increasing attentiveness to the fine-grained realities and entanglements of food, more-than-human becomings, and other-than-human beings as well as the situatedness of food in microbiosocial environments.
